# Reduced gene flow and bottleneck in the threatened giant armadillo (*Priodontes maximus*): implications for its conservation

**DOI:** 10.1590/1678-4685-GMB-2023-0252

**Published:** 2024-02-19

**Authors:** Nayra T. Rodrigues, Bruno H. Saranholi, Alexandre R. Inforzato, Leandro Silveira, Arnaud Leonard Jean Desbiez, Pedro M. Galetti

**Affiliations:** 1 Universidade Federal de São Carlos, Departamento de Genética e Evolução, São Carlos, SP, Brazil.; 2 Imperial College London, Department of Life Sciences, Ascot, United Kingdom.; 3 Instituto Onça-Pintada (IOP), Mineiros, GO, Brazil.; 4 Instituto de Conservação de Animais Silvestres (ICAS), Campo Grande, Mato Grosso do Sul, Brazil.; 5 Royal Zoological Society of Scotland (RZSS), Murrayfield, Edinburgh, United Kingdom.; 6 Instituto de Pesquisas Ecológicas (IPE), Nazaré Paulista, São Paulo, Brazil.

**Keywords:** Genetic diversity, habitat fragmentation, Xenarthra, Cingulata, animal Conservation

## Abstract

The progressive fragmentation and loss of habitats represent the main threats for endangered species, causing genetic consequences that may have potential implications for a population’s long-term persistence. Large mammals are the most affected species among vertebrates. The giant armadillo *Priodontes maximus* is a large South American mammal threatened species, showing nocturnal, solitary and fossorial behavior, occurring at low population densities, and its population dynamics are still poorly known. In this study, we carried out the first assessment of genetic variability and population genetic structure of the species, using a panel of 15 polymorphic microsatellites developed by high-throughput genome sequencing*.* The spatial Bayesian clustering, F_st_ and D_est_ results indicated the presence of two genetic clusters (K = 2) in the study area. These results suggest a reduction in gene flow between individuals inhabiting the Brazilian savanna (Cerrado) and the Pantanal wetlands, with the increased human-driven habitat modifications possibly contributing for this scenario. A bottleneck signal was detected in both populations, and a subpopulation structuring in the Cerrado may also be reflecting consequences of the extensive habitat modifications. Findings from this study provide important and useful information for the future maintenance of genetic diversity and long-term conservation of this flagship species.

## Introduction

Genetic diversity is a key element for the long-term persistence of a species (Frankel and Soulé, 1981), and its amount and distribution depend on several ecological and evolutionary factors, as well as the deleterious effects of human-driven habitat modifications (Frankel and Soulé, 1981; Frankham et al., 2002). A species with a wide geographic distribution can constitute a large and single panmictic population or different genetically connected populations (e.g., Epps et al., 2007; Marrotte et al., 2017; Saranholi et al., 2022). The connectivity among populations is highly associated to the dispersal capacity of the species and its response to barriers and habitat suitability (Kupfer et al., 2006). In turn, human-driven habitat loss and fragmentation have been threatening biodiversity (Storfer et al., 2010; Ahumada et al., 2011; Gibson et al., 2011; Haddad et al., 2015) by isolating populations and limiting gene flow (Gerlach and Musolf, 2000; Oklander et al., 2010; Haag et al., 2010; Saranholi *et al.*, 2017), as well as causing genetic diversity losses that may threaten long-term population persistence (Frankham *et al.*, 2002; Keyghobadi, 2007). 

Several species of mammals show wide geographic distributions in the continents where they occur, and genetic-based studies have been reporting different genetic diversity distribution patterns that can be explained by biogeographic features, dispersal capacity, and habitat adaptation (Clozato et al., 2015). However, due to their typically lower densities, low rates of population growth, and large home range requirements (Crooks et al., 2017), large mammals are the most affected species by habitat loss and fragmentation among vertebrates, resulting in loss of genetic diversity and in the isolation of their populations (reviewed in Lino et al., 2019). 

The giant armadillo *Priodontes maximus* Kerr, 1792 (Mammalia: Cingulata), the largest extant species of armadillo (Emmons and Feer, 1997; Carter et al., 2016; Desbiez et al., 2019a), is part of one of the most ancient lineages of placental mammals, the magna-order Xenarthra (Murphy et al., 2001). This is an ecologically very important species, acting as ecosystem engineers (Leite-Pitman et al., 2004; Desbiez and Kluyber, 2013; Aya-Cuero et al., 2017; Massocato and Desbiez, 2018; Di Blanco et al., 2020; Fontes et al., 2020). It has a distribution that extends over a large area of South America; however, it occurs in discontinuous populations and at low population densities (Cabrera, 1958; Desbiez *et al.*, 2020a; Meritt, 2006). 

In the southern part of its distribution in Brazil, the giant armadillo occurs in the Pantanal wetlands and in the Brazilian savanna (Cerrado). Wherever the species occurs, it is naturally rare, and it has become rarer due to the alterations and destruction of its habitat (Marinho-Filho and Medri, 2008; Ferraz et al., 2021), as is the case with the Cerrado domain, which is undergoing extensive modifications, due to the expansion of agriculture and cattle ranching. The giant armadillo presents a low population growth rate, with a litter size of one individual, prolonged parental care, and a three-year interbirth interval (Desbiez et al., 2019b). Furthermore, the age of sexual maturity is estimated to be between six and a half to eight years, with the longest generation time among the Xenarthra (Luba et al., 2020). Therefore, the loss of a single individual can have a significant impact on the population. Despite occurring in anthropized areas, several studies have shown that the giant armadillo depends mainly on native vegetation to survive, especially in its early stages of life (Vynne et al., 2011; Esteves et al., 2018; Desbiez *et al*., 2020c; Lemos et al., 2020). It is estimated that a population decline of at least 30% has already occurred over the past three generations (Anacleto *et al*., 2014), mainly due to anthropogenic actions such as habitat loss and fragmentation, hunting, roadkills, and illegal trafficking (Anacleto *et al*., 2014; Chiarello *et al*., 2015; Carter et al., 2016; Banhos et al., 2020). Currently, the giant armadillo is categorized as Vulnerable (A2cd) by the Red List of Threatened Species of the International Union for Conservation of Nature and Natural Resources (IUCN; Anacleto *et al*., 2014) and the Brazilian Institute for Biodiversity Conservation (ICMBio; Chiarello *et al*., 2015). 

Little is known on the population dynamics of these animals (Superina et al., 2014; Carter et al., 2016; Desbiez et al., 2020c , 2021a), and genetic studies have been hindered by the challenge of obtaining biological samples with quality DNA from wild populations (Benirschke and Wurster, 1969; Benirschke *et al*., 1969; Delsuc et al., 2002, 2003; Redi et al., 2005; Gibb et al., 2016). There is no available information on the population genetics of this species thus far. 

Luckily, a partnership with two long-term projects focused on monitoring giant armadillo and roadkills (Giant Armadillo Conservation Program and Anteaters and Highways Project, respectively), as well as a third project focused on ecology in a protected area (Parque Nacional das Emas) allowed us to obtain tissue samples of this rare animal for a very first population genetics study of this threatened species. In turn, microsatellites have been used for genetic studies on large number of metazoans worldwide during the two last decades (reviewed in mammals in Túnez et al., 2021), uncovering a wealth of ecological information concerning mammal species (Broquet et al., 2006; Beja-Pereira et al., 2009; Saranholi et al., 2023). Because microsatellites were absent for the giant armadillo, this study describes an initial panel of 15 microsatellites obtained through next-generation sequencing, which can be very helpful in studies of population genetics, as well as for assessing paternity and kinship. 

Considering the aforementioned ecological and behavioral characteristics, we hypothesize that (i) there is a reduced gene flow between the Pantanal wetlands and the Cerrado, resulting in population structuring between these two biomes. On the other hand, considering the extensive human-driven habitat modifications occurring especially in the Cerrado domain, we expect that (ii) gene flow can also be reduced between subpopulations within a biome. Finally, as it is well known that threatened species usually have small (or declining) and fragmented populations, which facilitate the loss of their genetic diversity (Frankham et al., 2002), we predict that (iii) loss of genetic variation may be occurring in giant armadillo, and the long-term persistence of these animals may be threatened. This study represents the first assessment of genetic diversity in populations of *P. maximus* and used a species-specific panel of microsatellites developed by us, using high throughput sequencing. Besides contributing to increasing the knowledge of the species, it brings new genetic data that may be useful for definitions of conservation strategies for this important endangered species. 

## Materials and Methods

### Ethical statements

Sample collection was performed in compliance with Brazilian legislation, under the SISBIO license number (53798-7) for sample collection permits and for SISGEN genetic material access authorization (A05D558), in addition to being approved by the Ethics Committee on Animal Use of the Federal University of São Carlos (CEUA/UFSCAR - 3597261118).

### Study area and samples

**Figure 1 -:**
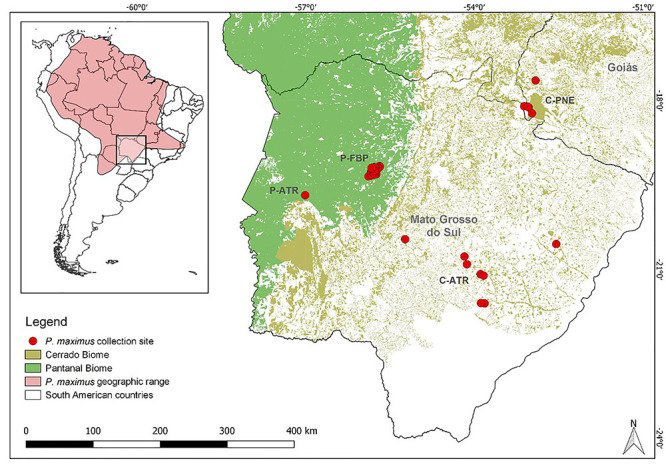
Geographic distribution of *P. maximus* and magnified area where sampling was carried out in the Pantanal and Cerrado biomes. P = Pantanal; C = Cerrado; P-ATR = Samples from roadkills in the Pantanal biome; P-FBP = Samples from Fazenda Baía das Pedras; C-ATR = Samples from roadkills in the Cerrado domain; C-PNE = Samples from Parque Nacional das Emas. Sources: *P. maximus* geographic range from IUNC (Anacleto *et al.*, 2014), and Biome limits obtained from IBGE (2019).

A total of 45 giant armadillo tissue samples was collected between 2010 and 2020 in two different Brazilian biomes: Pantanal and Cerrado (Figure 1). In the latter, four blood samples were collected from free-living animals in Parque Nacional das Emas (C-PNE) in the state of Goiás, and eight samples were obtained from roadkills along three highways located in the state of Mato Grosso do Sul - MS-040, MS-355, and BR-262/MS - provided by the Anteaters and Highways Project (C-ATR and P-ATR; https://www.tamanduabandeira.org/). In Pantanal, 32 ear tissue samples (21 from adult and 11 from sub-adult specimens) were obtained from animals monitored at Fazenda Baía das Pedras (P-FBP) located in the Pantanal wetlands, state of Mato Grosso do Sul, by the Giant Armadillo Conservation Program - coordinated by the Wild Animal Conservation Institute (https://www.icasconservation.org.br/projetos/tatucanastra/) - and one roadkill (BR-262/MS). Detailed information related to each sample is available in Table S1.

### DNA extraction and microsatellite development

DNA extraction from tissue samples was performed using the Phenol-Chloroform protocol (Sambrook and Russell, 2001). For the isolation of microsatellites, an aliquot of DNA with 468 ng in 15 μL (31.2ng/μL) was used. A single genomic library was prepared according to the standard protocol of the Illumina Nextera DNA Flex kit. Sequencing was performed from both ends (paired-end) using an Illumina HiSeq 2500 system. The search for microsatellites in the genome was performed using the MISA software (https://webblast.ipk-gatersleben.de/misa/), and the primers were designed using Primer3Plus (Untergasser et al., 2007). Considering only microsatellites larger than trinucleotides, 23,389 were found, comprising 5,372 trinucleotides, 15,244 tetranucleotides, 1,821 pentanucleotides, and 952 hexanucleotides.

For the final microsatellite panel, only simple microsatellites showing tetranucleotide motifs were selected for population validation, because they are more unstable than the complex microsatellites, and consequently can lead to a greater number of alleles (Chung et al., 1993; Pépin et al., 1995). We selected those microsatellites with eight or more repeats, favoring the chance that they are more mutable and consequently more polymorphic (Katti et al., 2001). The microsatellites should be in non-coding regions. Microsatellite sequences are deposited in the GenBank (https://www.ncbi.nlm.nih.gov/genbank/, Accession Number OM930795 - OM930809). 

### Microsatellite amplification and genotyping

For an initial population validation, 30 microsatellite primer pairs were synthesized with the addition of the M13 tail at the 5’ end of one of the primers (forward or reverse), according to Schuelke (2000). PCR amplifications were successful for 15 of the 30 loci tested, with PCR product size ranging from 209 to 278 bp (Table 1). For the amplification reactions, we used 1x GoTaq Buffer solution (Promega), 0.5-4.0 mM MgCl_2_ (Invitrogen), 0.2 mM of each dNTP, 0.1 μM of the primer with the M13 tail, 0.4 μM of the primer without the M13 tail, 0.4 μM of the M13 primer fluorescently labeled (FAN, PET, VIC, NED), 0.5 unit of GoTaq® DNA Polymerase, and 30 ng of DNA in a final reaction volume of 10 μl. PCR was performed in a Veriti 96 Well Thermal Cycler (Applied Biosystems). The amplification program consisted of initial denaturation at 94 °C for 5 min, followed by 35 cycles of 30 s at 94 °C, 45 s at the annealing temperature (AT, Table 1), 45 s at 72 ºC; 10 additional cycles starting at 94 °C for 30 s, 45 s at 53 °C (primer M13 annealing temperature), 45 s at 72 °C, and a final extension temperature of 72 °C for 20 min. 

**Table 1 -:** Characteristics of the 15 microsatellite loci developed and successfully amplified in *Priodontes maximus*. Loci names, forward and reverse primer sequences, motif with number of repeats, annealing temperature (AT), PCR product size, and GenBank accession number.

Loci	Primer sequence (5’-3’)		Motif	AT (ºC)	Product Size (pb)	Accession number
	Forward	Reverse				
Pmax02	CAAGCTCATGATCTGCACATGT	AGGATCCCAAGGTAACCTGA	(TCTA)_14_	62.0	258	OM930795
Pmax04	TCTAAGTTGTACATTGGTGTCTGT	TCTCCTCCCTCAGCATGACA	(TATC)_13_	64.0	214	OM930796
Pmax05	ACAGTAGGAACATCTTCACGAA	GCCCTACCAAAGCCATAATAGC	(TATC)_12_	60.0	245	OM930797
Pmax09	TCCCTGGGAGATACTCAAGGA	TCCACTTCCCTGTAGCTTGC	(TCTA)_12_	62.0	240	OM930798
Pmax11	ATCTCTTGTTTCTCTCAGAAGCT	TACAACCTGTGACTGCTGCA	(TAGA)_11_	64.0	223	OM930799
Pmax16	ACAATTTAGGACAGAAAAGGACAGA	CCCAAATACCCAGATCCTCCA	(TAGA)_10_	62.0	237	OM930800
Pmax17	TCACAGAAATAGAGGGTTCACAGA	AGTCAATCTTGCTTGTCTTCCA	(TAGA)_10_	60.0	209	OM930801
Pmax18	ACATCATTCTCCTCCCTGACA	TTGTCAGCCCACCCTACTTG	(GATA)_10_	62.0	210	OM930802
Pmax19	TCTGTGTTCTACCAGTCAAGCT	TGGTAACTCAATCCAGCAGTTCA	(CTAT)_10_	62.0	221	OM930803
Pmax21	AGTGCTCAAGGAACATGATGT	TCGACAGCACTGGTGATACA	(TAGA)_9_	60.0	278	OM930804
Pmax22	ACTTCACCAGCATTCACCAA	TGCTCAGCACCATGAAACAA	(GATA)_9_	62.0	256	OM930805
Pmax25	AGTGTTCAAGCAGCATGATGT	ACCCTTTATCCAGCTACCCAG	(GATA)_9_	60.0	209	OM930806
Pmax28	ACTCTTTTCTGCACACACTCT	GGCAGATAAGTAGCTGAGGCC	(ATAG)_8_	64.0	264	OM930807
Pmax29*	GGCTTGCCTCTTAGTCCACA	ACCCCAGTCTACCTCCTTCC	(TTCA)_8_	62.0	265	OM930808
Pmax30	GGCAAATTCATGGCAAGACTCT	AGAGAAAATGCAGAAAGATCACACT	(TATC)_8_	60.0	248	OM930809

* Removed from the subsequent analysis, since this locus was not polymorphic for the individuals analyzed.

Microsatellite amplification was confirmed by 2% agarose gel electrophoresis, and the PCR products were genotyped in an ABI 3730XL automated sequencer (Applied Biosystems). Fragment pattern and size analyses for genotype definition were performed using the microsatellite plugin in the Geneious 6.1.9 software (Kearse et al., 2012).

### Population genetic analyses

A panel of 14 polymorphic microsatellite loci was obtained (Table 1) and used for population genetic analyses. Genotypes were analyzed using Micro Checker 2.2.3 (Van Oosterhout et al., 2004) to detect null alleles and other genotyping errors. The existence of different genetic groups within the set of sampled individuals was analyzed through non-spatial and spatial models. A non-spatial Bayesian analysis was performed using the Structure software (Pritchard et al., 2000). We tested values of K ranging from 1 to 5 (number of sampled groups plus one: P-FBP, C-PNE, C-ATR, P-ATR) in 100 independent runs, using 200,000 Markov Chain Monte Carlo (MCMC) iterations, followed by 100,000 burning-in iterations. We performed the analyses using the Admixture Model with correlated allele frequencies. We repeated the analysis for two configurations: no *a priori* assignment to a given group, and with *a priori* information about the sampling biome (Cerrado vs. Pantanal; LOCPRIOR configuration); the latter configuration can infer the differences between groups of individuals with low genetic differentiation (Hubisz et al., 2009). To obtain the optimal value of K, we used the log-likelihood LnP (D/K) (Pritchard *et al.*, 2000) and the estimates of Delta-K (Evanno et al., 2005), determined through the online tool Structure Harvester (Earl and Vonholdt, 2012). The consensus individual assignment graph over the 100 independent runs was visualized in Cluster Markov Packager Across K - CLUMPAK (Kopelman et al., 2015). However, the Structure software may fail, in some situations, to detect the real number of clusters, due to its assumptions regarding the population models, so the use of different approaches is recommended (Jombart, 2008; Jombart *et al*., 2010). 

In this way, we also performed populational genetic structure analyses based on spatial models. This approach based on spatial components is recommended to improve the estimates of population structure because it is less affected by isolation by distance (François and Durand, 2010; Perez et al., 2018). We used two spatial genetic structure approaches to evaluate the distribution of genetic variability across space, Geneland (Guillot et al., 2005) and Spatial PCA - sPCA (Jombart, 2008). Both analyses are similar for including individual georeferenced multilocus genotypes to estimate the number of genetic clusters and genetic discontinuity. Geneland uses Bayesian inference and spatial location of samples, which provides further support for cluster analyses, even when cryptic patterns of population structuring occur (McManus et al., 2015), and can be especially useful in the case of sparse sampling (Ball et al., 2010). This analysis was performed using the Geneland 3.1.4 package (Guillot *et al*., 2005) available in the R software (R Core Team, 2018). Geneland was run assuming an uncorrelated model for allele frequencies. Although the correlated allele frequency model is more powerful in detecting subtle genetic differentiation, it seems more prone to algorithm instabilities, e.g., overestimating K values where isolation by distance occurs (Guillot, 2008). We performed 20 independent runs with K values ranging from 1 to 5, using 1,000,000 MCMC iterations and 1,000 thinning iterations. The final spatial model was run with K values ranging from one to the maximum number of clusters obtained in the initial runs, using 2,000,000 MCMC iterations and 1,000 thinning iterations in 10 independent runs. 

In turn, the sPCA is a multivariate approach that is free of Hardy-Weinberg equilibrium assumptions, in which the allele frequencies-based principal component score for each individual is multiplied by Moran’s I, a measure of spatial autocorrelation for that individual (Jombart, 2008). sPCA divides spatial autocorrelation into global and local structures, based on whether neighbors are positively or negatively spatially autocorrelated. Local structuring occurs when genetically similar individuals avoid mating with each other, whereas global structuring is expected in genetic clines or spatially distinct genetic groups. sPCA was performed using the adegenet R package (Jombart, 2008) with a distance-based connection network for sampling aggregate patterns. We tested for significant global and local structuring using a MCMC randomization test with 999 permutations (Jombart, 2008).

Following the detection of the genetic clusters, the Wright fixation index (F_ST_) was calculated using Arlequin v.3.5.2.2 (Excoffier and Lischer, 2010). As the traditional F_ST_ may present biases when estimated from highly polymorphic markers such as microsatellites (Jost, 2008; Heller and Siegismund, 2009), we also calculated the D differentiation index (D_est_) proposed by Jost (2008), using the DEMEtics package (Gerlach et al., 2010), implemented in R (R Core Team, 2018).

We performed a spatial autocorrelation analysis (SAA) in GenAlEx version 6.5 (Peakall and Smouse, 2012), to analyze the effect of isolation by distance (IBD) on population differentiation. The method evaluates the genetic distance between pairs of individuals in distance classes. The statistical significance (p < 0.05) for the spatial autocorrelation coefficients (r) was obtained through 9,999 permutations and 9,999 bootstraps. The distance classes were variable and divided into 2 km, 4 km, 6 km, 8 km, 10 km, 15 km, 25 km, 250 km, 350 km, and 450 km.

Genetic diversity parameters were estimated for the genetic groups found in the population structuring analyses. Deviations from the Hardy-Weinberg equilibrium and linkage disequilibrium between individual pairs of loci were evaluated using GENEPOP (Raymond and Rousset, 1995) with 10,000 repetitions, correcting the p-values according to the Bonferroni procedure (Rice, 1989). The number of alleles (N_A_), the number of effective alleles (A_E_), as well as expected (H_E_) and observed (H_O_) heterozygosity, were calculated in GenAlex v6.5 (Peakall and Smouse, 2012). By using FSTAT 2.9.3.2 (Goudet, 2001), we calculated the inbreeding coefficient (F_IS_), and p-values for excess and deficit of heterozygotes and allelic richness (R_A_). The polymorphic information content (PIC) for each locus was evaluated using the CERVUS software (Kalinowski et al., 2007). Effective population size (N_E_) was estimated based on linkage disequilibrium (Waples, 2006) using NeEstimator v2.1 (Do et al., 2014) assuming minimum allele frequency of rare alleles equal to 0.05 and 0.01. 

To detect genetic evidence of population decline, we used the Bottleneck software (Cornuet and Luikart, 1996; Piry *et al*., 1999). The values were obtained by simulations under three mutation models: the infinite allele model (IAM), stepwise mutation model (SMM), and two-phase mutation (TPM), accepting 70% and 90% of the stepwise mutation (Piry *et al*., 1999), using 1,000 iterations. The Wilcoxon test was applied to determine the statistical significance of the results (p<0.05), which is appropriate for analyses with less than 20 loci (Piry *et al*., 1999). Populations exhibiting significant excess of heterozygotes would be considered to have experienced a recent genetic bottleneck. To exclude any sample size bias, we used HybridLab 1.0 (Nielsen et al., 2006) to have the same number of individuals for each genetic group from simulated individuals using the allele frequencies of the base population, and then we ran Bottleneck with the same parameters as described above. 

## Results

Of the 30 microsatellite primer pairs synthesized for validation, 15 loci were successfully amplified (Table 1), and 14 loci showed polymorphism among the giant armadillo individuals studied (Table 2), resulting in a very informative microsatellite-panel (PIC >0.5; Table 2). Since the locus Pmax29 was not polymorphic, the subsequent genetic analyses were performed using a set of the 14 polymorphic microsatellite loci.

**Table 2 -:** Parameters of genetic diversity for each genetic cluster, based on 14 microsatellite loci. N = number of individuals analyzed; N_A_ = number of alleles per locus; R_A_ = allele richness; A_E_ = effective number of alleles; PIC = polymorphic information content; HWE = p-value of Fisher's exact test for adherence to Hardy-Weinberg equilibrium (α = 0.05); H_O_ = observed heterozygosity; H_E_ = expected heterozygosity; F_IS_ = inbreeding coefficient. Significance levels of F_IS_ values for p < 0.00357; P_S_ = smaller F_IS_ values; P_L_ = larger F_IS_ values.

	Loci	N	N_A_	R_A_	A_E_	PIC	HWE	H_O_	H_E_	F_IS_	P_S_	P_L_
Cerrado Cluster (N=12)	Pmax02	12	3	2.990	2.380	0.502	0.911	0.667	0.580	-0.107	0.410	0.839
	Pmax04	11	5	4.818	4.246	0.724	0.791	0.727	0.764	0.096	0.732	0.489
	Pmax05	12	3	3.000	2.796	0.57	0.187	0.833	0.642	-0.257	0.335	0.828
	Pmax09	12	4	3.740	2.526	0.541	0.818	0.583	0.604	0.078	0.689	0.567
	Pmax11	11	4	3.974	3.227	0.633	0.669	0.818	0.690	-0.139	0.210	0.921
	Pmax16	10	4	3.900	3.279	0.633	0.212	0.500	0.695	0.328	0.964	0.125
	Pmax17	12	3	2.990	2.133	0.468	0.187	0.417	0.531	0.257	0.982	0.089
	Pmax18	12	2	2.000	2.000	0.375	0.248	0.333	0.500	0.371	0.992	0.192
	Pmax19	9	4	4.000	2.282	0.512	0.725	0.778	0.562	-0.333	0.092	1.000
	Pmax21	11	3	3.000	2.988	0.591	0.580	0.545	0.665	0.226	0.950	0.142
	Pmax22	12	4	3.748	2.420	0.535	0.410	0.833	0.587	-0.384	0.025	1.000
	Pmax25	12	3	3.000	2.642	0.55	0.860	0.583	0.622	0.105	0.703	0.532
	Pmax28	12	4	3.686	1.704	0.386	0.970	0.500	0.413	-0.168	0.317	1.000
	Pmax30	11	4	3.971	2.444	0.547	0.692	0.636	0.591	-0.029	0.682	0.635
	Mean	-	3.571	3.487	2.648	0.540	-	0.625	0.603	-	-	-
	Total	-	50	-	-	-	-	-	-	0.044	0.575	0.428
Pantanal Cluster (N=33)	Pmax02	33	3	2.968	2.380	0.510	0.957	0.576	0.589	0.023	0.596	0.532
	Pmax04	32	7	5.618	4.808	0.762	0.034	0.781	0.805	0.029	0.735	0.428
	Pmax05	33	4	3.450	2.489	0.540	0.671	0.545	0.607	0.104	0.864	0.321
	Pmax09	33	3	2.808	2.310	0.471	1.000	0.576	0.576	0.000	0.610	0.557
	Pmax11	33	5	3.701	2.789	0.573	1.000	0.697	0.651	-0.071	0.353	0.789
	Pmax16	32	6	5.377	4.491	0.743	0.552	0.813	0.790	-0.029	0.489	0.685
	Pmax17	32	3	2.636	2.190	0.438	0.611	0.625	0.552	-0.134	0.289	0.828
	Pmax18	33	4	3.241	2.565	0.539	0.240	0.667	0.620	-0.077	0.375	0.742
	Pmax19	33	6	5.036	2.892	0.624	0.787	0.788	0.664	-0.189	0.035	0.992
	Pmax21	33	3	2.991	2.767	0.562	0.267	0.576	0.648	0.114	0.857	0.246
	Pmax22	33	4	3.050	1.659	0.364	0.196	0.394	0.403	0.023	0.725	0.489
	Pmax25	32	3	2.994	2.602	0.546	0.721	0.719	0.625	-0.152	0.185	0.896
	Pmax28	33	5	3.740	2.979	0.603	0.091	0.606	0.675	0.103	0.878	0.246
	Pmax30	33	4	3.256	2.461	0.532	0.732	0.606	0.603	-0.005	0.557	0.628
	Mean	-	5.452	3.633	2.813	0.557	-	0.640	0.629	-	-	-
	Total	-	60	-	-	-	-	-	-	-0.018	0.296	0.707

### Population Genetic Structuring

The non-spatial Structure clustering analysis based on the LnP value indicated K = 1 as the most likely K for both the no LOCPRIOR and LOCPRIOR models. In contrast, the results yielded by the method proposed by Evanno et al. (2005) indicated K = 2 as the most probable value for the no LOCPRIOR (Figure S1) and LOCPRIOR (Figure S2). However, this method does not allow for the calculation of Delta K when K = 1. In the individual assignment graph, the individuals have similar probabilities of belonging to both clusters, suggesting that no population structuring could be detected by these methods (Figure S3). 

**Figure 2 -:**
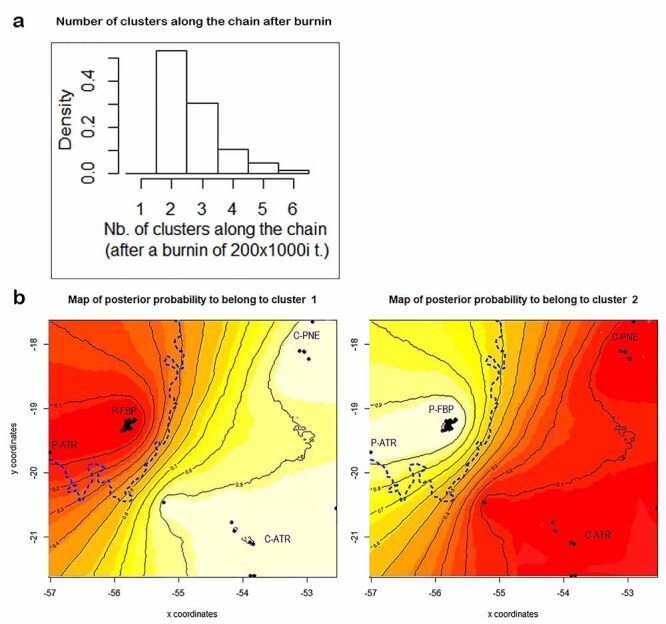
Graph with the most probable number of clusters (K=2) provided by the Geneland package (a) and posterior probability maps for spatial assignment of *Priodontes maximus* (b). Spatial grouping suggests two distinct genetic clusters throughout the geographic area surveyed. The dashed line corresponds to the approximated boundary between the Pantanal and Cerrado biomes. Black dots represent the locations of the individuals. Higher probabilities of belonging to the cluster are represented by the colors yellow and white. P = Pantanal; C = Cerrado; P-ATR = Samples from roadkills in the Pantanal; P-FBP = Samples from Fazenda Baía das Pedras; C-ATR = Samples from roadkills in the Cerrado; C-PNE = Samples from Parque Nacional das Emas.

**Figure 3 -:**
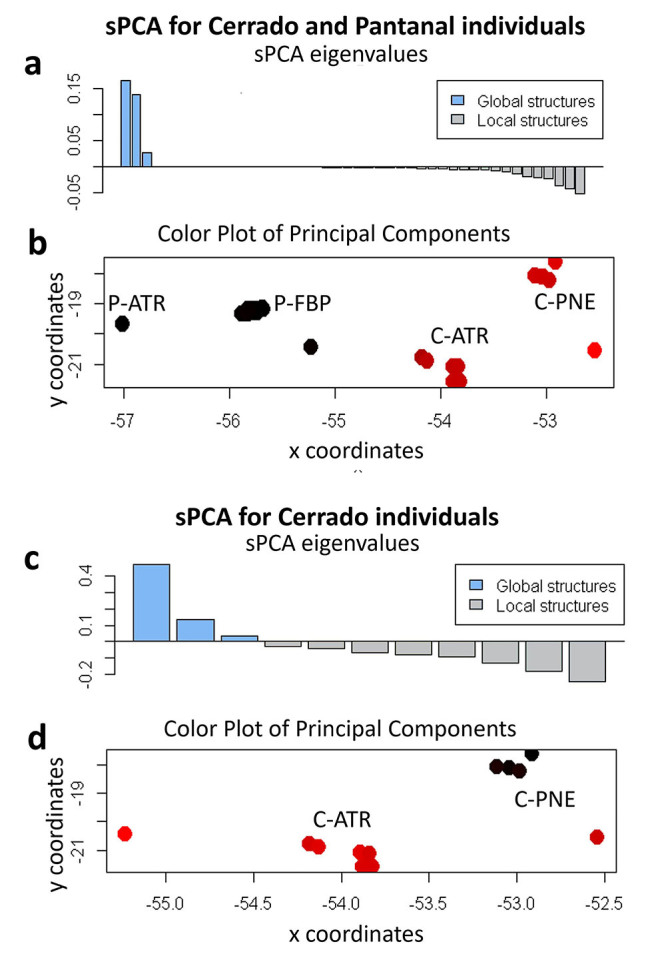
Genetic structure assessed by sPCA of *Priodontes maximus*. (a) and (c) Barplot showing that spatially meaningful genetic variance in the dataset is contained in the first three positive axes of the global structure (indicated by the blue bars), while, comparatively, very little variance is present in local structure (indicated by the grey bars) for all individuals and only for Cerrado, respectively. (b) and (d) The results show the first Principal Component (PC) and the respective mappings of cluster membership (colors represent the membership probability for two clusters) for all individuals and only for Cerrado, respectively.

On the other hand, the spatial Bayesian analysis performed in Geneland (Guillot et al., 2005) identified the presence of two genetic clusters, separating the sampled individual groups from Pantanal (P-FBP + P-ATR) and Cerrado (C-PNE + C-ATR) with posterior probabilities of 90% (Figure 2). It is worth noting that the same pattern was observed in the sPCA (Figure 3), which indicates that most variation occurs in the global structure (Figure 3a), resulting in two genetic clusters (Figure 3b). The presence of these two genetic clusters was also confirmed by the significant results for Wright’s fixation index (F_ST_ = 0.0253, p = 0.0021) and Jost differentiation index (D_est_ = 0.03830, p = 0.006).

**Figure 4 -:**
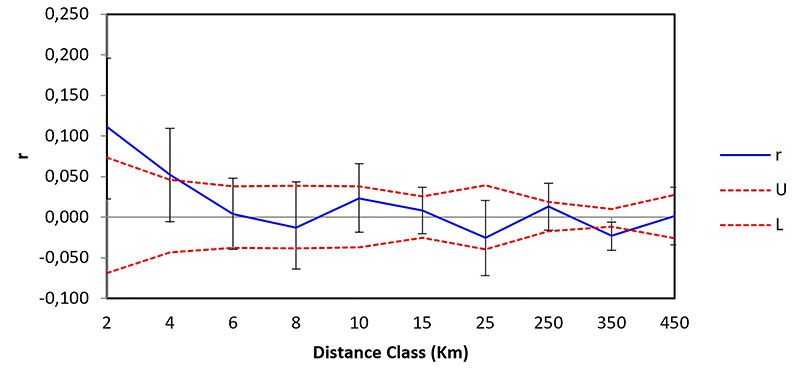
Spatial autocorrelation for *Priodontes maximus.* The graphs show the genetic correlation coefficient (r) as a function of the geographic distance between the defined spatial distance classes. Red dashed lines represent the upper (U) and lower (L) bounds of the null hypothesis (no spatial structure) based on 10,000 random permutations. Error bars represent 95% confidence intervals on r based on 1,000 bootstraps.

Spatial autocorrelation analysis (SAA) showed a significant positive correlation only for the first (0-2 km; r = 0.114 with p = 0.004) and second (2-4 km; r = 0.054 with p = 0.011) distance classes, indicating that individuals within both classes are more genetically similar than individuals that are more spatially distant. The *x*-intercept of *r* was between 6 and 8 km (Figure 4). Indeed, a subpopulation structure was detected by sPCA analysis within the Cerrado cluster, in agreement with the findings indicating that spatially closer individuals are genetically more similar (Figure 3d).

### Genetic diversity

No evidence of null alleles was found, and no locus is either in linkage disequilibrium (LD) after the Bonferroni correction (p = 0.0035) or outside the expected values of Hardy-Weinberg equilibrium (p > 0.0035) for both populations identified in the spatial analysis (Pantanal and Cerrado). It is worth mentioning that significant linkage disequilibrium was observed between two loci (Pmax22 and Pmax25) after the Bonferroni correction (p = 0.00357) when all the samples were treated as a single population. 

The mean observed and expected heterozygosity values did not differ significantly between the Cerrado (Ho = 0.625, He = 0.603) and Pantanal (Ho = 0.640, He = 0.629) populations (Table 2). No significant F_IS_ values were found for both populations. The mean allelic richness (R_ACerrado_= 3.487; R_APantanal_= 3.633) and numbers of effective alleles (A_ECerrado_= 2.648; A_EPantanal_ = 2.813) were similar between both populations. The average PIC values (> 0.50) showed that the set of microsatellite loci used was highly informative. 

The effective population size (N_E_), calculated using all individuals (45), ranged from 42.1 (95% CI = 28.8 - 67.9) to 59.3 (95% CI = 38.4 - 109.7), assuming minimum frequency of rare alleles as 0.05 and 0.01, respectively.

The Wilcoxon significance test found a significant excess of heterozygotes in the Cerrado (p < 0.05) for n = 12 individuals (collected data) using the IAM and TPM accepting both 70% and 90% of SMM. When using simulated genotypes (n = 33), a significant excess of heterozygotes was found using all three mutation models tested (Table 3). In turn, in the Pantanal (n=33) we found a significant excess of heterozygotes (p < 0.05) using IAM, TPM with 70% and 90% of SMM, and SMM (Table 3).

**Table 3 -:** P-values for excess and/or deficit of heterozygotes generated by the Wilcoxon test. IAM = infinite allele mutation model; SMM = stepwise mutation model; TPM = two-phase mutation model, accepting 70% and 90% of the stepwise mutation model. Results for n = 12 (collected data) and n = 33 (simulated genotypes) in Cerrado and n = 33 in Pantanal (collected data). * significant p-values p < 0.05.

	12				33			
	IAM	TPM		SMM	IAM	TPM		SMM
		70%	90%			70%	90%	
Cerrado	0.00031*	0.00671*	0.03381*	0.06763	0.00003*	0.00021*	0.00131*	0.01227*
Pantanal	-	-	-	-	0.00006*	0.00076*	0.02472*	0.12061

## Discussion

Results from this study indicate a probable gene flow restriction between Pantanal and Cerrado populations, and a potential loss of genetic variation for giant armadillos within the study area, corroborating our initial predictions. The 14 microsatellite loci developed in this study were successfully amplified and showed a very informative mean PIC value, according to the classical work of Botstein et al. (1980), which makes them very useful for population genetic analyses. Samples for which a few loci (1 to 4) were not successfully amplified turned out to be from roadkills. It is notoriously difficult to obtain a large amount of high-quality DNA in such cases, due to post-mortem DNA degradation under high environmental temperatures and UV radiation (Rodríguez-Castro et al., 2017; Amarilla-Stevens et al., 2023), which are typical in the region studied.

Spatial Bayesian analysis (K=2) suggests a reduction in gene flow between giant armadillos inhabiting the Pantanal and the Cerrado. With high assignment values, this analysis allocated all individuals sampled in the Cerrado domain to a single population, separated from those allocated in Pantanal. The presence of two genetic clusters (Pantanal and Cerrado) was reinforced by the F_ST_ and D_est_ results. To some extent, these results are expected, considering the already established fact that the giant armadillo occurs at low densities, and in discontinuous populations (Cabrera, 1958; Meritt, 2006; Desbiez et al., 2020a, 2020c). The detection of linkage disequilibrium between two loci (Pmax22 and Pmax25) when all samples were analyzed as a single population corroborates these findings, and is consistent with the Admixture linkage disequilibrium (ALD) that arises when two separate populations are mixed (Pfaff et al., 2001). The disagreement between the optimal K-values found by the non-spatial (Structure) and spatial (Geneland and sPCA) analyses may be due to the assumptions made in the population models used in the former which, in some cases, may fail to detect the actual number of clusters (Jombart, 2008; Jombart *et al.*, 2010); for example, in cases where populations have lower levels of genetic divergence, with F_ST_ values < 0.03 (Latch et al., 2006; Waples and Gaggiotti, 2006), as observed in our study between the Pantanal and Cerrado populations. Additionally, where cryptic patterns of population structuring may occur, the spatial model used by both Geneland and sPCA offers greater support to clustering analyses (e.g., McManus et al., 2015), and these analyses are also particularly useful in situations where sampling is sparse (Ball et al., 2010), as is the case of our study. Further studies with a larger and spatially wider sampling set - as well as using a SNP panel largely distributed in the genome - are encouraged for confirming the population genetic structuring observed.

This scenario of genetic population differentiation between Cerrado and Pantanal may have a historical explanation, considering the different characteristics of the biomes (vegetation, climate, altitude), as well the discontinuity already reported between giant armadillo populations (Cabrera, 1958; Meritt, 2006; Desbiez et al., 2020a,c). However, the increased environmental degradation already reported in the surveyed region (Ferraz et al., 2021) can also be a contributing factor, as it severely limits the movement of individuals between Cerrado and Pantanal. The conversion of land within Cerrado into agricultural and pasture areas is primarily responsible for the loss of permeability and functional connectivity (Sugai et al., 2014), and the large reduction and fragmentation of viable habitats for the giant armadillo in this domain may promote the further isolation of its populations (Ferraz *et al*., 2021). 

The significant positive correlation found for distances of up to 4 km obtained by the SAA analysis indicates that individuals spatially closer are more genetically similar than those who are spatially more distant. These findings suggest an isolation by distance model at least for short distances, which could be modified by a break in the typical IBD clinal allele frequency pattern (Ruiz-Gonzalez et al., 2015) at longer distances. A quite similar situation was reported in tapir along an Atlantic Forest corridor (Saranholi et al., 2022). The authors found a significant spatial autocorrelation at short distances, suggesting that gene flow in tapir was mostly IBD-regulated, but a break in the clinal IBD pattern resulted in population structuring likely induced by gene flow barriers promoted by human-driven landscape modifications (Saranholi *et al*., 2022). It is worth noting that the geographical distance between the Cerrado and Pantanal sampling sites (C-ATR x P-FBP and C-PNE x P-FBP) being shorter than the distance between the two Cerrado sampling sites (C-ATR and C-PNE), reinforce that a break in a potential clinal IBD pattern may be promoting population structuring between Pantanal and Cerrado. In addition, the *x*-intercept at 8 km in the SSA analysis indicates a tendency to a limited range of dispersion (Janecka et al., 2017; Maciel et al., 2019) of giant armadillo in the studied area, corroborating the subpopulation structuring observed in the Cerrado. However, considering that a median home range of 25 km^2^ (or 2500 ha) for adult giant armadillos has been reported for the individuals in Pantanal (Desbiez et al., 2020a), this small dispersion range (8 km) suggested by the spatial autocorrelation analysis still could benefit from a wider sampling, for a more precise decision.

The genetic diversity represented by allelic richness was very similar in both populations studied. It is important to highlight that the mean allelic richness observed here (R_ACerrado_ = 3.487; R_APantanal_ = 3.633) for the giant armadillo was lower than that found for other, non-threatened Xernathrans, such as *Chaetophractus vellerosus* (R_A_ = 15; Nardelli et al., 2016) and *Dasypus novencinctus* (R_A_ = 12.6; Arteaga et al., 2012). Endangered species that have suffered a reduction in their population size have likely lost many of their alleles, so most of them have lower allele richness than related non-endangered species (Frankham et al., 2002), and this may well be the case observed here in the giant armadillo. The number of effective alleles was also low in both populations (A_ECerrado_ = 2.648; A_EPantanal_ = 2.813), indicating that the number of alleles that actually contribute to genetic diversity is lower than the number of total alleles found. This result can stem from the small effective population size, which prevents the retention of all alleles at high frequencies in both populations (Kimura and Crow, 1964). This is what is expected for a highly endangered species. 

Our results suggest that there has been a recent bottleneck in giant armadillo. Although all components of genetic diversity are affected by a reduction in population size, bottlenecks have a greater immediate effect on allele number than on heterozygosity, causing heterozygosity excess at selectively neutral loci (Nei et al., 1975; Allendorf, 1986; Spencer et al., 2000). Large losses of heterozygosity are more likely if the bottleneck lasts for several generations, or if the recovery of the population after the bottleneck is slow (Leberg, 1992). Since the excess of heterozygotes observed when the population undergoes a recent reduction can be detected during 0.25 to 2.5 x 2Ne generations, our results demonstrate that the species suffered a population reduction more ancient than the recent three-generations reduction suggested by Anacleto *et al*. (2014). In fact, the population reduction persists to current generations, as a result of targeted hunting, road collisions, as well as the continued loss and fragmentation of the habitats where the species occurs (Chiarello *et al*., 2015; Alho et al., 2019; Banhos et al., 2020; Ferraz et al., 2021). 

Certain consequences of reduced population may not be observed until several generations after the bottleneck (Price and Hadfield, 2014). Long generation times and lifespans can function as intrinsic buffers against loss of genetic diversity (Hailer et al., 2006), resulting in delayed detection of genetic diversity loss. The giant armadillo is suspected to have a natural life expectancy of more than 20 years (Desbiez et al., 2020b , 2021b), a generation time of 8 years (Luba et al., 2020) and a low population growth rate, with a litter size of one and extended parental care (Desbiez *et al*., 2019b). These biological characteristics may explain the putative slow reduction in the heterozygosity found here. 

Despite occurring in anthropized areas, the giant armadillo survives by feeding mainly on native vegetation (Vynne et al., 2011; Esteves et al., 2018; Desbiez et al., 2020c; Lemos et al., 2020; Ferraz et al., 2021), and this aspect is crucial for the conservation of the species. However, our results suggest that the increased human-driven habitat modification, particularly in the Cerrado domain, may have genetically impacted the giant armadillo, leading to the reduced gene flow observed between Pantanal and Cerrado, and to the bottleneck detected in both populations. The subpopulation structuring detected in the Cerrado, increasing the level of discontinuity between populations, gives credit to the suggestion that genetic consequences of habitat modifications can already be felt, and threaten local populations of giant armadillos. The bottlenecks and reduction in gene flow may be acting in synergy to decrease both genetic diversity and population capability to persist. The expansion of fully protected areas, creation of corridors, road passages, and other conservation actions would be recommended, and could be crucial for mitigating the endangerment and boosting species persistence not only for the giant armadillo, but other local species as well. Given the current conservation status of the giant armadillo, it is imperative that its genetic diversity and population structure should be assessed throughout the species distribution, so that effective conservation actions may be planned and brought to fruition, in order to ensure its long-term viability.

## Supplementary Material

The following online material is available for this article:

Table S1 - Location of samples used in the study.

Figure S1 - Delta K (a) and L(K) (b) values without prior information (no LOCPRIOR) obtained from the Bayesian clustering analysis performed in Structure.

Figure S2 - Delta K (a) and L(K) (b) values with prior information (LOCPRIOR) obtained from the Bayesian clustering analysis performed in Structure.

Figure S3 - Results of the Bayesian clustering analysis performed in Structure, showing the percentage of membership of each individual to each cluster.
