# Supplementary material for: Reduced gene flow and bottleneck in the threatened giant armadillo (Priodontes maximus): implications for its conservation
Source: Genet Mol Biol. 2024 Feb 19;47(1):e20230252. doi: 10.1590/1678-4685-GMB-2023-0252 (PMC10917080; doi:10.1590/1678-4685-GMB-2023-0252)
Supplement: Table S1 - [file 1415-4757-GMB-47-1-e20230252-s1.pdf]

Genetics and Molecular Biology
https://doi.org/10.1590/1678-4685-GMB-2023-0252

Supplementary Material to "Reduced gene flow and bottleneck in the threatened giant armadillo (*Priodontes maximus*): implications for its conservation"

Table S1 - Location of samples used in the study.

ID	Latitude	Longitude	Biome	Sampling Site
**RK457**	-20.4619	-55.2317	Cerrado	C-ATR
**RK615**	-20.769	-54.1776	Cerrado	C-ATR
**RK1086**	-20.9073	-54.1335	Cerrado	C-ATR
**RK1089**	-21.6035	-53.8179	Cerrado	C-ATR
**RK1287**	-21.0838	-53.8905	Cerrado	C-ATR
**RK1294**	-21.1081	-53.8365	Cerrado	C-ATR
**RK1302**	-20.5479	-52.5445	Cerrado	C-ATR
**RK1301**	-21.5978	-53.8825	Cerrado	C-ATR
**A**	-18.1156	-53.0357	Cerrado	C-PNE
**B**	-17.6391	-52.912	Cerrado	C-PNE
**C**	-18.0971	-53.1139	Cerrado	C-PNE
**D**	-18.2224	-52.9755	Cerrado	C-PNE
**RK429**	-19.6792	-57.0097	Pantanal	P-ATR
**TC-01**	-19.2952	-55.7704	Pantanal	P-FBP

ID	Latitude	Longitude	Biome	Sampling Site
**TC-02**	-19.2956	-55.7594	Pantanal	P-FBP
**TC-04**	-19.332	-55.8522	Pantanal	P-FBP
**TC-05**	-19.3446	-55.8847	Pantanal	P-FBP
**TC-06**	-19.3006	-55.8139	Pantanal	P-FBP
**TC-07**	-19.3138	-55.7826	Pantanal	P-FBP
**TC-08**	-19.2402	-55.8022	Pantanal	P-FBP
**TC-09**	-19.1629	-55.689	Pantanal	P-FBP
**TC-10**	-19.2913	-55.8303	Pantanal	P-FBP
**TC-11**	-19.293	-55.8187	Pantanal	P-FBP
**TC-12**	-19.2281	-55.7966	Pantanal	P-FBP
**TC-13**	-19.2291	-55.7973	Pantanal	P-FBP
**TC-14**	-19.2802	-55.8403	Pantanal	P-FBP
**TC-15**	-19.2523	-55.827	Pantanal	P-FBP
**TC-16**	-19.3067	-55.7446	Pantanal	P-FBP
**TC-17**	-19.3232	-55.8322	Pantanal	P-FBP
**TC-18**	-19.3084	-55.7731	Pantanal	P-FBP
**TC-19**	-19.2536	-55.8329	Pantanal	P-FBP
**TC-20**	-19.1717	-55.6796	Pantanal	P-FBP
**TC-21**	-19.2157	-55.7858	Pantanal	P-FBP
**TC-22**	-19.1961	-55.7059	Pantanal	P-FBP
**TC-23**	-19.2826	-55.7878	Pantanal	P-FBP

ID	Latitude	Longitude	Biome	Sampling Site
**TC-24**	-19.2052	-55.7772	Pantanal	P-FBP
**TC-25**	-19.181	-55.7803	Pantanal	P-FBP
**TC-26**	-19.2091	-55.7327	Pantanal	P-FBP
**TC-27**	-19.231	-55.7773	Pantanal	P-FBP
**TC-28**	-19.2067	-55.8075	Pantanal	P-FBP
**TC-29**	-19.1834	-55.7645	Pantanal	P-FBP
**TC-30**	-19.2165	-55.8023	Pantanal	P-FBP
**TC-31**	-19.2509	-55.776	Pantanal	P-FBP
**TC-32**	-19.2073	-55.8167	Pantanal	P-FBP
**TC-33**	-19.1926	-55.8302	Pantanal	P-FBP
