# Supplementary material for: Reduced gene flow and bottleneck in the threatened giant armadillo (Priodontes maximus): implications for its conservation
Source: Genet Mol Biol. 2024 Feb 19;47(1):e20230252. doi: 10.1590/1678-4685-GMB-2023-0252 (PMC10917080; doi:10.1590/1678-4685-GMB-2023-0252)
Supplement: Figure S1 - [file 1415-4757-GMB-47-1-e20230252-s2.pdf]

Genetics and Molecular Biology
https://doi.org/10.1590/1678-4685-GMB-2023-0252

Supplementary Material to "Reduced gene flow and bottleneck in the threatened giant armadillo (*Priodontes maximus*): implications for its conservation"

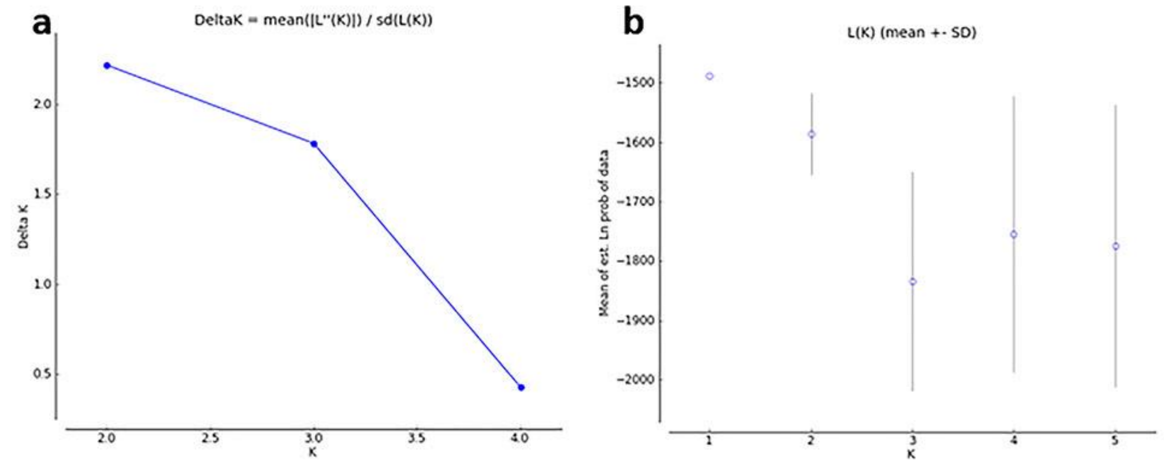

Figure S1 - Delta K (a) and L(K) (b) values without prior information (no LOCPRIOR) obtained from the Bayesian clustering analysis performed in Structure.
